# Thermal micropolar and couple stresses effects on peristaltic flow of biviscosity nanofluid through a porous medium

**DOI:** 10.1038/s41598-022-20320-6

**Published:** 2022-09-28

**Authors:** Aya M. Ismael, Nabil T. Eldabe, Mohamed Y. Abou zeid, Sami M. El Shabouri

**Affiliations:** 1 Department of Mathematics, Faculty of Science, Ain Shams University, Abbasiya, Egypt; 2 Department of Mathematics, Faculty of Education, Ain Shams University, Roxy, Cairo, Egypt

**Keywords:** Fluid dynamics, Applied mathematics

## Abstract

The main aim of the current study is to analyze couple stresses effects on MHD peristaltic transport of a micropolar non-Newtonian nanofluid. The fluid flows through a porous media between two horizontal co-axial tubes. The effects of radiation, chemical reaction, viscous and ohmic dissipation are considered. The inner tube is solid and uniform, while the outer tube has a sinusoidal wave traveling down its wall. The governing equations have been simplified using low-Reynolds number and long wave-length approximations, thus a semi-analytical solutions have been obtained using the homotopy perturbation method. Numerical results for the behaviors of the axial velocity, microrotation velocity, temperature and nanoparticles concentration with the physical parameters are depicted graphically through a set of graphs. Furthermore, the values of the skin friction coefficient, Nusselt and nano Sherwood numbers are computed and presented graphically through some draws. Moreover, the trapping phenomenon is discussed throughout a set of figures. The present study is very important in many medical applications, as the gastric juice motion in the small intestine when an endoscope is inserted through it. Further, gold nanoparticles are utilized in the remedy of cancer tumor.

## Introduction

The fluid which contain nanometer-sized particles is called nanofluid. These fluids are colloidal suspensions engineered of nanoparticles in a base fluid, which are typically made of oxides, carbides, metals, or carbon nanotubes. Abouzeid^1^ discussed the effect of Cattaneo-Christov heat flux of biviscosity nanofluid on MHD flow between two rotating disks through a porous media. The influences of heat generation, chemical reaction and uniform magnetic field on the flow of non-Newtonian nanofluid down a vertical cylinder is studieded by El-Dabe and Abouzeid^2^. El-Dabe and Abouzeid^3^ analyzed the influences of Joule heating, thermal-diffusion with thermal radiation and internal heat generation of a non-Newtonian fluid on peristaltic flow using Jeffery model. The importance of velocity second slip model on peristaltic pumping of non-Newtonian fluid in existence of induced magnetic field and double-diffusivity convection in nanofluids is explained by Akram et al.^4^. Abouzied^5^ studied analytically the couple stresses influences on MHD peristaltic transport of a non-Newtonian Jeffery nanofluid. Analytically study with heat transfer the motion of power-law nanofluid under the effect radiation, internal heat generation and viscous dissipation are studied by Ismael et al.^6^. Ouaf et al.^7^ studied the influences of slip velocity condition and entropy generation through a porous medium on MHD Jeffery nanofluid flow in a channel with peristalsis. Heat transfer aspects of a heated Newtonian viscous fluid and the flow properties are studied mathematically inside a vertical duct having elliptic cross section and sinusoidally fluctuating walls with single wall carbon nanotubes by Akhtar et al.^8^. Eldabe et al.^9^ studied Dufour effects and Soret on peristaltic flow in a uniform symmetric channel with wall properties of non-Newtonian magnetohydrodynamic (MHD) nanofluid. Recently, there are many papers related to nanofluid over different surfaces^10–17^.

Micropolar fluids consider as a special case of classical model established Navier–Stokes, is call polar fluids with microstructure with nonsymmetric stress tensor. Eldabe et al.^18^ discussed the influence of the induced magnetic field which contain gyrotactic microorganisms on Eyring-Powell nanofluid Al2O3 motion through the boundary-layer. Mixed convention and uniform inclined magnetic field influences with heat transfer on non-Newtonian micropolar nanofluid Al2O3 flow are discussed by Eldabe et al.^19^. Akhtar et al.^20^ discussed mathematically the physics of peristaltic flow with mass and heat transfer effects in elliptic duct with taking in consideration a non-Newtonian Casson fluid model. Theoretical analysis of combined mass and heat transfer over an oscillatory inclined porous plate in unsteady mixed convection flow of micropolar fluid in a homogenous porous medium with radiation absorption, Joule dissipation and heat source is discussed by Shamshuddin et al.^21^. Eldabe and Abouzeid^22^ studied the peristaltic transport of non-Newtonian micropolar fluid. Many results of the micropolar are studied in these articles^23–28^.

The couple stress is a fluid related to fluids which contain particles randomly oriented and rigid suspended in a viscous medium. The electro-osmotic peristaltic flow is studied of a couple stress fluid bounded in micro-channel asymmetric inclined by Reddy^29^. Abouzeid^30^ presented an analytical discussion for couple stresses impacts of a non-Newtonian Jeffery nanofluid on MHD peristaltic transport. Under the suspension of small particles the peristaltic induced motion of couple stress fluid have been explained by Bhatti^31^. Eldabe et al.^32^ discussed with mass and heat transfer the peristaltic motion of a coupled stress fluid in a channel with compliant walls through a porous medium. In a non-uniform rectangular duct the peristaltic flow of couple stress liquid is studied by Ellahi^33^. Recently, there are different papers that studied the couple stress fluid^34–42^.

The fundamental target of this study focusses on describing the impacts of couple-stress theories as well as thermal micropolar properties on peristaltic motion of non-Newtonian nanofluid. The fluid is flowing through a porous media between two co-axial horizontal cylinders. In addition, the effects of both viscous and Ohmic dissipation and chemical reaction are also included. Moreover, the mathematical intricacy of our study can be alleviating by applying the long wavelength and low Reynold’s number presumptions. These non-linear equations are analytically disbanded by applying the conventional perturbation method together with homotopy analytical method up to the second order. Numerical results for the behaviors of the axial velocity, microrotation velocity, temperature and nanoparticles concentration with the physical parameters are depicted graphically through a set of graphs. Furthermore, the values of the skin friction coefficient, Nusselt and nano Sherwood numbers are computed and presented graphically through some draws. Moreover, the trapping phenomenon is discussed throughout a set of figures. The influences of diverse physical parameters on the various distributions are analyzed numerically and displayed through a set of graphs. The current study is very significant in several medical applications, like the gastric juice motion in the small intestine when an endoscope is inserted through it. The endoscope has many clinical applications. Hence, it is considered to be a very significant tool used in determining real reasons responsible for many problems in the human organs in which fluid is transported by peristaltic pumping, such as the stomach, small intestine, etc. Also, gold nanoparticles are used in the remedy of cancer tumor.

## Mathematical description

**Figure 1:**
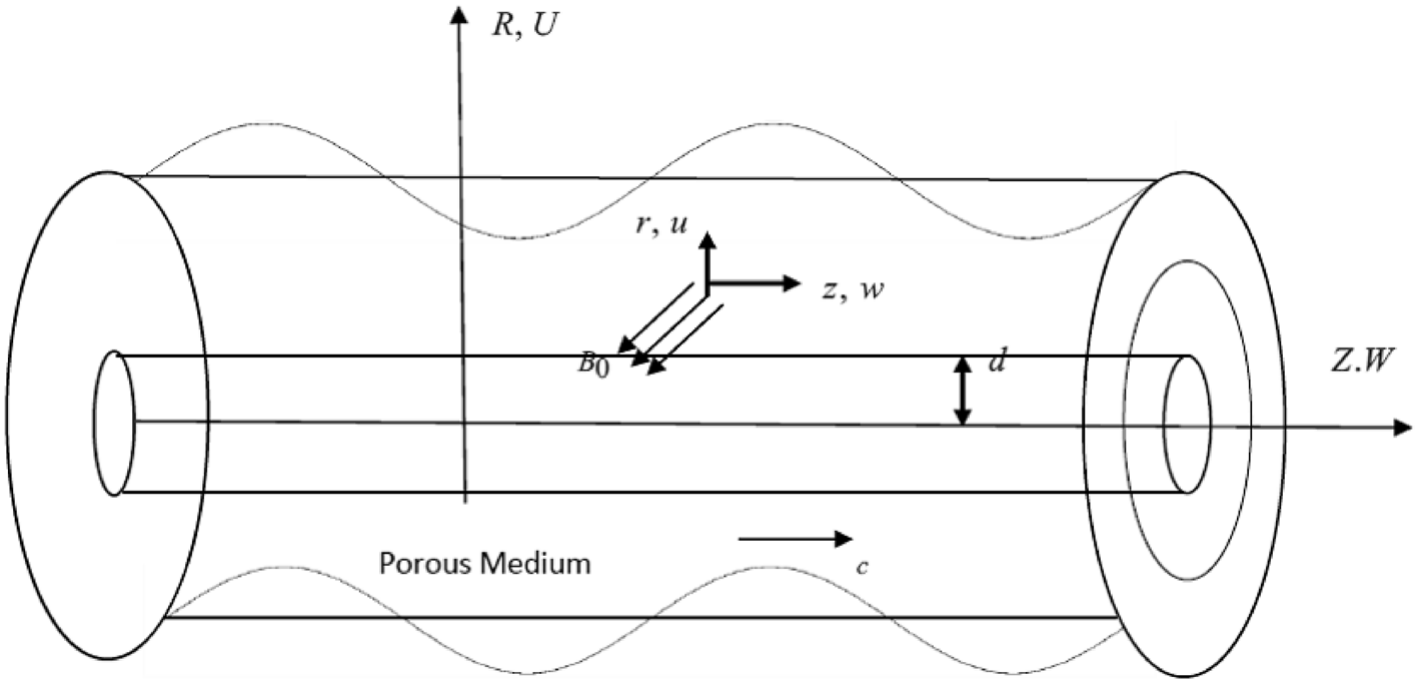
Diagram of fluid flow.

A two-dimensional unsteady peristaltic flow of an incompressible non-Newtonian micropolar nanofluid are considered. The fluid flows through a porous media between two co-axial tubes under the effects of radiation, chemical reaction, viscous and ohmic dissipation. The inner tube is solid and uniform, while the outer tube has a sinusoidal wave traveling down its wall with a constant speed $c$. The system is stressed by a magnetic field of a strength $B_{0} ,$ see Fig. 1.

Fluid model is studied in cylindrical coordinate system $\left(r,\theta ,z\right)$. Channel wall has a mathematical description as

1 $$r_{1} = 0.3\;d,$$

2 $$r_{2} = H = d + b{\kern 1pt} {\kern 1pt} \sin \frac{2\pi }{\lambda }(z - ct),$$

Let the respective velocity components in the axial and the radial direction in the fixed frame are W and U, respectively. For the unsteady two dimensional flow, the velocity, micro-rotation velocity, temperature and nanoparticles components may be written as

$$\begin{gathered} \underline{V} = (U(R,Z),0,W(R,Z)),\quad \underline{N} = (0,N_{\theta } ,0) \hfill \\ T = T(R,Z)\quad {\text{and}}\quad f = f(R,Z) \hfill \\ \end{gathered}$$

A wave frame (r, z) moving with velocity c away from the fixed frame (R, Z) by the transformation:

3 $$\begin{array}{*{20}l} {r = R,} \hfill & {z = Z - ct,} \hfill \\ {u = U,} \hfill & {w = W - c} \hfill \\ \end{array}$$

The governing equations of the motion of this model can be represented as[^5,22^]:

4 $$\frac{\partial u}{{\partial r}} + \frac{u}{r} + \frac{\partial w}{{\partial z}} = 0,$$

5 $$\begin{aligned} \rho_{f} \left( {u\frac{\partial u}{{\partial r}} + w\frac{\partial u}{{\partial z}}} \right) = & - \frac{\partial p}{{\partial r}} + \left( {\mu_{f} \left( {1 + \gamma^{ - 1} } \right) + k_{1} } \right)\left( {\frac{{\partial^{2} u}}{{\partial r^{2} }} + \frac{1}{r}\frac{\partial u}{{\partial r}} - \frac{u}{{r^{2} }} + \frac{{\partial^{2} u}}{{\partial z^{2} }}} \right) - \sigma B_{0}^{2} u \\ & \quad - \frac{{\mu_{f} }}{{k^{*} }}u - k_{1} \frac{{\partial N_{\theta } }}{\partial z} - \eta \nabla^{4} u, \\ \end{aligned}$$

6 $$\begin{aligned} \rho_{f} \left( {u\frac{\partial w}{{\partial r}} + w\frac{\partial w}{{\partial z}}} \right) = & - \frac{\partial p}{{\partial z}} + \left( {\mu_{f} \left( {1 + \gamma^{ - 1} } \right) + k_{1} } \right)\left( {\frac{{\partial^{2} w}}{{\partial r^{2} }} + \frac{1}{r}\frac{\partial w}{{\partial r}} - \frac{{\partial^{2} w}}{{\partial z^{2} }}} \right) - \sigma B_{0}^{2} w \\ & \quad - \frac{{\mu_{f} }}{{k^{*} }}w + k_{1} \frac{1}{r}\frac{{\partial (r{\kern 1pt} N_{\theta } )}}{\partial r} - \eta \nabla^{4} w, \\ \end{aligned}$$

7 $$\rho j\left( {u\frac{{\partial {\kern 1pt} N_{\theta } }}{\partial r} + w\frac{{\partial {\kern 1pt} N_{\theta } }}{\partial z}} \right) = - 2k_{1} N_{\theta } + \gamma^{*} \left( {\frac{\partial }{\partial r}\left( {\frac{1}{r}\frac{{\partial (r{\kern 1pt} N_{\theta } )}}{\partial r}} \right) + \frac{{\partial^{2} N_{\theta } }}{{\partial z^{2} }}} \right) + k_{1} \left( {\frac{{\partial {\kern 1pt} u}}{\partial z} - \frac{{\partial {\kern 1pt} w}}{\partial r}} \right),$$

8 $$\begin{aligned} \rho {\kern 1pt} c_{p} \left( {u\frac{\partial T}{{\partial r}} + w\frac{\partial T}{{\partial z}}} \right) = & k_{f} \left( {\frac{{\partial^{2} T}}{{\partial r^{2} }} + \frac{1}{r}\frac{\partial T}{{\partial r}} + \frac{{\partial^{2} T}}{{\partial z^{2} }}} \right) + \left( {\mu_{f} (1 + \gamma^{ - 1} ) + k_{1} } \right)\left[ {2\left( {\frac{\partial u}{{\partial r}}} \right)^{2} + 2\left( {\frac{\partial w}{{\partial z}}} \right)^{2} + \left( {\frac{{\partial {\kern 1pt} u}}{\partial z} + \frac{{\partial {\kern 1pt} w}}{\partial r}} \right)^{2} } \right] \\ & \quad + (\rho {\kern 1pt} c)_{p} {\kern 1pt} \left[ {D_{B} \left( {\frac{\partial T}{{\partial r}}\frac{\partial f}{{\partial r}} + \frac{\partial T}{{\partial z}}\frac{\partial f}{{\partial z}}} \right) + \frac{{D_{T} }}{{T_{0} }}\left[ {\left( {\frac{\partial T}{{\partial r}}} \right)^{2} + \left( {\frac{\partial T}{{\partial z}}} \right)^{2} } \right]} \right] \\ & \quad + 2k_{1} \left[ {N_{\theta }^{2} - N_{\theta } \left( {\frac{{\partial {\kern 1pt} u}}{\partial z} - \frac{{\partial {\kern 1pt} w}}{\partial r}} \right)} \right] - \frac{1}{r}\frac{{\partial (r{\kern 1pt} q_{r} )}}{\partial r} + \sigma B_{0}^{2} {\kern 1pt} (u^{2} + (w + c)^{2} ) \\ \end{aligned}$$

9 $$\left( {u\frac{\partial f}{{\partial r}} + w\frac{\partial f}{{\partial z}}} \right) = D_{B} \left( {\frac{{\partial^{2} f}}{{\partial r^{2} }} + \frac{1}{r}\frac{\partial f}{{\partial r}} + \frac{{\partial^{2} f}}{{\partial z^{2} }}} \right) + \frac{{D_{T} }}{{T_{0} }}\left( {\frac{{\partial^{2} T}}{{\partial r^{2} }} + \frac{1}{r}\frac{\partial T}{{\partial r}} + \frac{{\partial^{2} T}}{{\partial z^{2} }}} \right) - A(f - f_{0} ),$$

The boundary conditions are given by:

10 $$u = 0,\;w = 0,\;T = T_{0} ,\;N_{\theta } = N_{{\theta_{{{\kern 1pt} 0}} }} ,\;f = f_{0} \quad at{\kern 1pt} \quad r = r_{1}$$

11 $$u = - c\frac{\partial H}{{\partial z}},\;w = - c,\;T = T_{1} {\kern 1pt} ,\;N_{\theta } = N_{{\theta {\kern 1pt}_{1} }} ,\;f = f_{1} \quad at\quad r = r_{2}$$

By using Rosseland approximation^43^, the radiative heat flux is given by

$$q_{r} = \frac{{ - 4\sigma^{*} }}{{3k_{R} }}\frac{{\partial T^{4} }}{\partial r}$$

where $k_{R}$ is the mean absorption coefficient and $\sigma^{*}$ is the Stefan Boltzmann constant. The temperature within the flow taking sufficiently small such that $T^{4}$ may considered as a linear function of temperature. This is accomplished by expanding $T^{4}$ in a Taylor series about $T_{1}$ and neglecting the higher-order terms, one gets

$$T^{4} \approx 4T_{1}^{3} T - 3T_{1}^{4}$$

Dimensionless quantities can be written as

12 $$\begin{aligned} & r^{*} = \frac{r}{d},\;z^{*} = \frac{z}{\lambda },\;u^{*} = \frac{\lambda }{{c{\kern 1pt} d}}u,\;w^{*} = \frac{w}{c},\;\delta = \frac{d}{\lambda },\;\overline{\gamma } = \frac{{\gamma^{*} }}{{d^{2} {\kern 1pt} \mu_{f} }},\;h = \frac{H}{d}, \\ & T^{*} = \frac{{T - T_{0} }}{{T_{1} - T_{0} }},\;f^{*} = \frac{{f - f_{0} }}{{f_{1} - f_{0} }},\;p^{*} = \frac{{d^{2} }}{{c{\kern 1pt} {\kern 1pt} \mu_{f} {\kern 1pt} \lambda }}p,\;Nt = \frac{{\varpi D_{T} (T_{1} - T_{0} )}}{{c{\kern 1pt} d{\kern 1pt} T_{0} }}, \\ & Nb = \frac{{\varpi D_{B} (f_{1} - f_{0} )}}{{c{\kern 1pt} d{\kern 1pt} }},\;N_{\theta }^{*} = \frac{d}{c}N_{\theta } ,\;j^{*} = \frac{j}{{d^{2} {\kern 1pt} }},\;\varepsilon = \frac{b}{d}\;t^{*} = \frac{c}{\lambda }t,\;\beta = \frac{{k_{1} }}{{{\kern 1pt} \mu_{f} }}, \\ & \varpi = \frac{{(\rho {\kern 1pt} c)_{p} }}{{(\rho {\kern 1pt} c)_{f} }},\;\Pr = \frac{{(\rho {\kern 1pt} c)_{f} {\kern 1pt} c{\kern 1pt} d}}{{k_{f} }},\;\frac{1}{\alpha } = \sqrt {\frac{{\mu_{f} }}{\eta }} d \\ \end{aligned}$$

Here $Da = \frac{{k^{*} }}{{d^{2} }}$ is Darcy number, $R_{e} = \frac{{\rho {\kern 1pt} c{\kern 1pt} d}}{{\mu_{f} }}$ is Reynolds number, $\Pr = \frac{{\mu_{f} {\kern 1pt} c{\kern 1pt}_{p} }}{{k_{f} }}$ is Prandtl number, $Ec = \frac{{c^{2} }}{{c_{p} {\kern 1pt} (T_{1} - T_{0} )}}$ is Eckert number, $M = \frac{{\sigma B_{0}^{2} d^{2} }}{{\mu_{f} }}$ is the magnetic field parameter and $R = \frac{{4{\kern 1pt} \sigma^{*} {\kern 1pt} T_{1}^{3} }}{{k_{f} {\kern 1pt} k_{R} }}$ is the radiation parameter.

In this these transformations, after applying $\delta \ll 1$ and neglecting the star mark, the system of equations takes the form:

13 $$\frac{\partial u}{{\partial r}} + \frac{u}{r} + \frac{\partial w}{{\partial z}} = 0$$

14 $$\frac{\partial p}{{\partial r}} = 0$$

15 $$\frac{\partial p}{{\partial z}} = \left( {(1 + \gamma^{ - 1} ) + \beta } \right){\kern 1pt} \left( {\frac{{\partial^{2} w}}{{\partial r^{2} }} + \frac{1}{r}\frac{\partial w}{{\partial r}}} \right) + \beta \left( {\frac{{\partial N_{\theta } }}{\partial r} + \frac{{N_{\theta } }}{r}} \right) - \left( {M + \frac{1}{Da}} \right)w - \alpha^{2} \nabla^{4} w$$

16 $$2{\kern 1pt} \beta N_{\theta } + \beta \frac{\partial w}{{\partial r}} = \overline{\gamma }\left[ {\frac{{\partial^{2} N_{\theta } }}{{\partial r^{2} }} + \frac{1}{r}\frac{{\partial N_{\theta } }}{\partial r} - \frac{{N_{\theta } }}{{r^{2} }}} \right]$$

17 $$\begin{aligned} & \left( {1 + \frac{4}{3}R} \right)\left( {\frac{{\partial^{2} T}}{{\partial r^{2} }} + \frac{1}{r}\frac{\partial T}{{\partial r}}} \right) + Ec\Pr \left( {(1 + \gamma^{ - 1} ) + \beta } \right)\left( {\frac{{\partial {\kern 1pt} w}}{\partial r}} \right)^{2} {\kern 1pt} + Nt\Pr \left( {\frac{{\partial {\kern 1pt} T}}{\partial r}} \right)^{2} + Nb\,Pr\left( {\frac{\partial f}{{\partial r}}} \right)\left( {\frac{\partial T}{{\partial r}}} \right) \\ & \quad + 2{\kern 1pt} \beta Ec\Pr \left[ {N_{\theta }^{2} + N_{\theta } \frac{{\partial {\kern 1pt} w}}{\partial r}} \right] + \sigma {\kern 1pt} B_{0}^{2} (w + 1)^{2} + Ec\Pr Mw^{2} = 0 \\ \end{aligned}$$

18 $$\left( {\frac{{\partial^{2} f}}{{\partial r^{2} }} + \frac{1}{r}\frac{\partial f}{{\partial r}}} \right) + \frac{Nt}{{Nb}}\left( {\frac{{\partial^{2} T}}{{\partial r^{2} }} + \frac{1}{r}\frac{\partial T}{{\partial r}}} \right) - \delta_{1} f = 0$$

At the wall, we will take the components of the couple stress tensor to be zero. Thus, the boundary conditions (10) and (11) in dimensionless will be written as

19 $$u = 0,\;w = 0,\;\frac{{\partial^{2} w}}{{\partial r^{2} }} - \frac{{\eta^{\prime}}}{r}\frac{\partial w}{{\partial r}} = 0\;T = f = 1,\;N_{\theta } = 0,\quad at{\kern 1pt} \quad r = r_{1} = 0.3$$

20 $$u = - c\frac{\partial h}{{\partial z}},\;w = - 1,\;\frac{{\partial^{2} w}}{{\partial r^{2} }} - \frac{{\eta^{\prime}}}{r}\frac{\partial w}{{\partial r}} = 0,\;T = f = 0{\kern 1pt} ,\;N_{\theta } = 1,\quad at\quad r = r_{2} = 1.2$$

## Method of solution

The homotopy perturbation method (HPM) is used to obtain an approximate solutions of the ordinary differential and the nonlinear partial differential equations. It combines between the advantages of the homotopy analysis method and the classical perturbation method. The homotopy Method is employing with an artificial parameter $P \in \left[ {0,1} \right]$, which is known as the homotopy parameter. Consequently, throughout this method, the small parameter can be put as a coefficient of any term of the problem.

Therefore, we use the homotopy perturbation method to solve these equations

21 $$\begin{aligned} H(p,w) = & (1 - p)[L_{1} (w) - L_{1} (w_{0} )] + p{\kern 1pt} (L_{1} (w) \\ & \quad + \frac{1}{{\alpha^{2} }}\left( {\frac{\partial p}{{\partial z}} - \left( {\left( {1 + \gamma^{ - 1} } \right) + \beta } \right)\left( {\frac{{\partial^{2} w}}{{\partial r^{2} }} + \frac{1}{r}\frac{\partial w}{{\partial r}}} \right) - \beta \left( {\frac{{\partial N_{\theta } }}{\partial r} + \frac{{N_{\theta } }}{r}} \right) + \left( {M + \frac{1}{Da}} \right)w} \right) \\ \end{aligned}$$

22 $$H(p,N_{\theta } ) = (1 - p)[L_{2} (N_{\theta } ) - L_{2} (N_{{\theta_{0} }} )] + p\left( {L_{2} (N_{\theta } ) - \frac{1}{{\overline{\gamma }}}\left( {2\beta N_{\theta } + \beta \frac{\partial w}{{\partial r}}} \right) - \frac{{N_{\theta } }}{{r^{2} }}} \right)$$

23 $$\begin{aligned} H(p,T) = & (1 - p)[L_{2} (T) - L_{2} (T_{0} )] + p{\kern 1pt} (L_{2} (T) + \left( {\frac{3}{3 + 4R}} \right)\left( {EcPr\left( {\left( {1 + \gamma^{ - 1} } \right) + \beta } \right)\left( {\frac{\partial w}{{\partial r}}} \right)^{2} } \right. \\ & \quad \left. { + Nt\Pr \left( {\frac{{\partial {\kern 1pt} T}}{\partial r}} \right)^{2} + Nb\Pr \left( {\frac{\partial f}{{\partial r}}\frac{\partial T}{{\partial r}}} \right) + 2\beta Ec\Pr \left[ {N_{\theta }^{2} + N_{\theta } \frac{{\partial {\kern 1pt} w}}{\partial r}} \right] + \sigma B_{0}^{2} (w + 1)^{2} + Ec\Pr Mw^{2} } \right) \\ \end{aligned}$$

24 $$H(p,f) = (1 - p)[L_{2} (f) - L_{2} (f_{0} )] + p\left( {L_{2} (f) + \frac{Nt}{{Nb}}\left( {\frac{{\partial^{2} T}}{{\partial r^{2} }} + \frac{1}{r}\frac{\partial T}{{\partial r}}} \right) - \delta_{1} f} \right)$$

with $L_{1} = \frac{{\partial^{4} }}{{\partial r^{4} }} + \frac{2}{r}\frac{{\partial^{3} }}{{\partial r^{3} }} - \frac{1}{{r^{2} }}\frac{{\partial^{2} }}{{\partial r^{2} }} + \frac{1}{{r^{3} }}\frac{\partial }{\partial r}$ and $L_{2} = \frac{{\partial^{2} }}{{\partial r^{2} }} + \frac{1}{r}\frac{\partial }{\partial r}$ as the linear operator. The initial guess $w_{0}$,$T_{0}$, $f_{0}$ and $N_{{\theta {\kern 1pt}_{0} }}$ can be written as $w_{0} = \frac{{r^{{1 + \eta^{\prime}}} - r_{1}^{{1 + \eta^{\prime}}} }}{{r_{1}^{{1 + \eta^{\prime}}} - r_{2}^{{1 + \eta^{\prime}}} }},\quad T_{0} = f_{0} = \frac{{Log[r] - Log[r_{2} ]}}{{Log[r_{1} ] - Log[r_{2} ]}},\quad N_{{\theta {\kern 1pt}_{0} }} = \frac{{Log[r] - Log[r_{1} ]}}{{Log[r_{2} ] - Log[r_{1} ]}}$ (25).

Now, it is assumed that:

26 $$(w,T,N_{\theta } ,f) = (w_{0} ,T_{0} ,N_{{\theta {\kern 1pt}_{0} }} ,f_{0} ) + p(w_{1} ,T_{1} ,N_{{\theta {\kern 1pt}_{1} }} ,f_{1} ) + \cdots .$$

The solutions of axial velocity, temperature, the micro-rotation velocity and nanoparticles concentration are:

27 $$\begin{aligned} w(r,z) = & \frac{{r^{{1 + \eta^{\prime}}} - r_{1}^{{1 + \eta^{\prime}}} }}{{r_{1}^{{1 + \eta^{\prime}}} - r_{2}^{{1 + \eta^{\prime}}} }} + a_{1} r^{4} + a_{2} r^{3} Log[r] + a_{3} r^{3} + r^{{\eta^{\prime}}} \left( {a_{4} r^{3} + a_{5} r^{5} } \right) \\ & \quad + a_{6} r^{2} + a_{7} r^{2} Log[r] + a_{8} Log[r] + a_{9} \\ \end{aligned}$$

28 $$\begin{aligned} T(r,z) = & \frac{{Log[r] - Log[r_{2} ]}}{{Log[r_{1} ] - Log[r_{2} ]}} + r^{{2 + 2\eta^{\prime}}} (a_{10} + a_{11} r^{2} ) + r^{{2 + \eta^{\prime}}} \left( {a_{12} r + a_{13} Log[r] + a_{14} } \right) \\ & \quad + a_{15} Log[r]^{2} + a_{16} r^{2} Log[r]^{2} + a_{17} r^{2} Log[r] + a_{18} r^{2} + a_{19} Log[r] + a_{20} \\ \end{aligned}$$

29 $$\begin{aligned} N_{\theta } (r,z) = & \frac{{Log[r] - Log[r_{1} ]}}{{Log[r_{2} ] - Log[r_{1} ]}} + a_{21} r^{{2 + \eta^{\prime}}} + a_{22} r^{2} Log[r] + a_{23} Log[r]^{3} \\ & \quad + a_{24} Log[r]^{2} + a_{25} r^{2} + a_{26} Log[r] + a_{27} \\ \end{aligned}$$

30 $$f(r,z) = \frac{{Log[r] - Log[r_{2} ]}}{{Log[r_{1} ] - Log[r_{2} ]}} + a_{28} r^{2} + a_{29} r^{2} Log[r] + a_{30} Log[r] + a_{31}$$

The skin friction coefficient $\tau_{\omega }$ may be introduced as:

31 $$\tau_{\omega } = \left[ {\left( {\left( {1 + \gamma^{ - 1} } \right) + \beta } \right)\frac{\partial w}{{\partial r}} + \beta N_{\theta } } \right]_{{r = r_{2} }} ,$$

The local Nusselt number $Nu$ may be written as:

32 $$Nu = \left. {\frac{\partial T}{{\partial r}}} \right|_{{r = r_{2} }} ,$$

The nano-Sherwood number $Sh$ may be defined as:

33 $$Sh = \left. {\frac{\partial f}{{\partial r}}} \right|_{{r = r_{2} }}$$

## Result and discussion

In this paper, we assumed that long wavelength and low-Reynolds number approximations to simplify the system of the nonlinear partial differential equations which describe the motion of our problem, i.e., the parameter δ assumed to be very small. Then the equations are solved by using the homotopy perturbation method. The effects of the physical parameter of the problem on the solution are discussed numerically and illustrated graphically.

The default values of problem related parameters are taken as:

$$\begin{aligned} & Pz = 1,\;\gamma = 0.7,\;\alpha = 0.05,\;\beta = 10,\;M = 10,\;Da = 2,\;R = 1, \\ & Ec = 20,\;Pr = 1.5,\;Nt = 5.5,\;Nb = 2.5, \;\overline{\gamma } = 1.2,\;\eta {\prime } = 1,\; r1 = 0.3,\; r2 = 1.2,\delta_{1} \; = 0.4. \\ \end{aligned}$$

**Figure 2:**
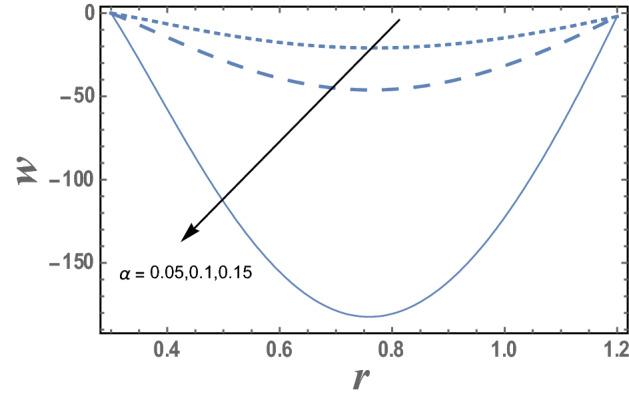
The variation of the axial velocity is plotted with r, for the different values of couple stress fluid parameter α and for a system has particular values Pz = 1, γ = 0.7, α = 0.05, $\beta$ = 10, M = 10, Da = 2, R = 1, Ec = 20, Pr = 1.5, Nt = 5.5, Nb = 2.5, $\overline{\gamma }$ = 1.2, $\eta^{\prime}$ = 1, ${r}_{1}$=0.3, ${r}_{2}$=1.2, $\delta_{1}$ = 0.4.

**Figure 3:**
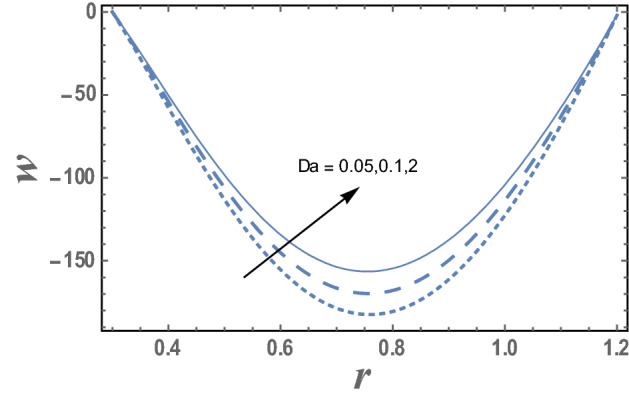
The variation of the axial velocity is plotted with r, for the different values of Darcy number Da and for a system has particular values Pz = 1, γ = 0.7, α = 0.05, $\beta$ = 10, M = 10, Da = 2, R = 1, Ec = 20, Pr = 1.5, Nt = 5.5, Nb = 2.5, $\overline{\gamma }$ = 1.2, $\eta^{\prime}$ = 1, ${r}_{1}$=0.3, ${r}_{2}$=1.2, $\delta_{1}$ = 0.4.

Figures 2 and 3 explain the couple stress fluid parameter α and the Darcy number Da on the axial velocity w, respectively. As can be seen from these figures that the axial velocity increases as α increases while it decreases as Da increase. Must also be noted that for each value of both α and Da, the axial velocity has a minimum value, i.e., w decreases as r increases till a minimum value, which it increases, and all minimum values occur at r = 0.75.

**Figure 4:**
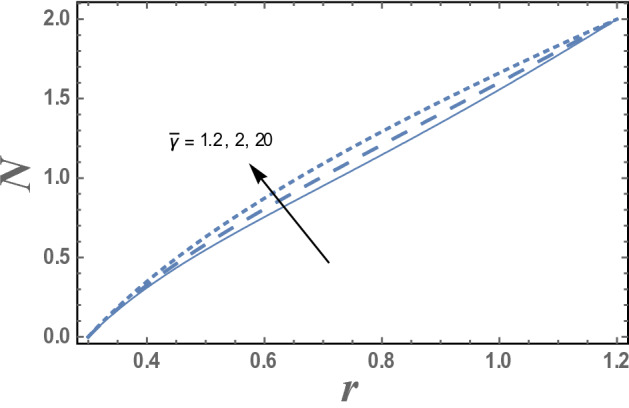
The variation of the micro-rotation velocity N is plotted with r, for different values of micro-rotation parameter $\overline{\gamma }$ and for a system has particular values Pz = 1, γ = 0.7, α = 0.05, β = 10, M = 10, Da = 2, R = 1, Ec = 20, Pr = 1.5, Nt = 5.5, Nb = 2.5, $\eta^{\prime}$ = 1, ${r}_{1}$ = 0.3, ${r}_{2}$ = 1.2, $\delta_{1}$ = 0.4.

**Figure 5:**
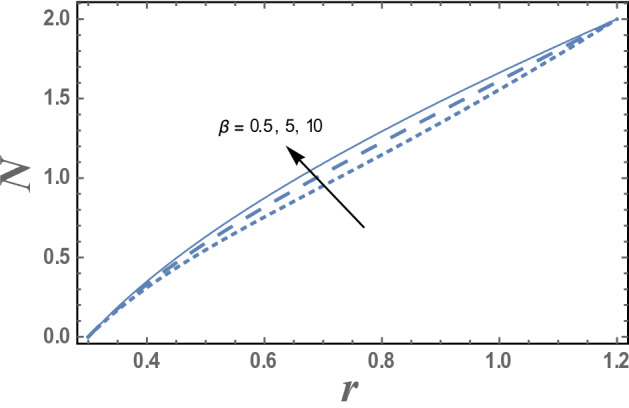
The variation of the micro-rotation velocity N is plotted with r, for different values of the dimensionless viscosity ratio β and for a system has particular values. Pz = 1, γ = 0.7, α = 0.05, M = 10, Da = 2, R = 1, Ec = 20, Pr = 1.5, Nt = 5.5, Nb = 2.5, $\overline{\gamma }$ = 1.2, $\eta^{\prime}$ = 1, ${r}_{1}$ = 0.3, ${r}_{2}$ = 1.2, $\delta_{1}$ = 0.4.

**Figure 6:**
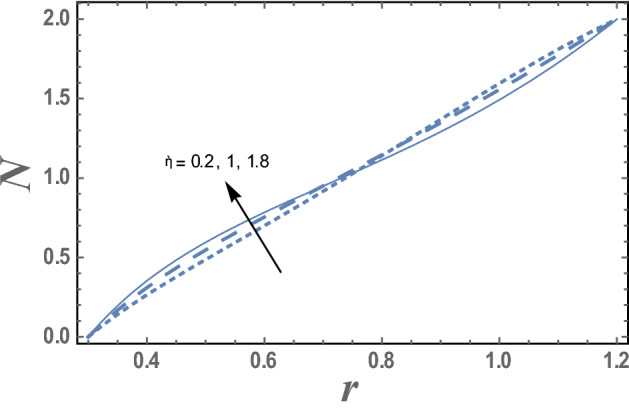
The micro-rotation velocity N is plotted with r, for different values of couple stress constant $\eta^{\prime}$ and for a system has particular values Pz = 1, γ = 0.7, α = 0.05, $\beta$ = 10, M = 10, Da = 2, R = 1, Ec = 20, Pr = 1.5, Nt = 5.5, Nb = 2.5, $\overline{\gamma }$ = 1.2, $\eta^{\prime}$ = 1, ${r}_{1}$=0.3, ${r}_{2}$=1.2, $\delta_{1}$ = 0.4.

The effect of the micro-rotation parameter $\overline{\gamma }$, the dimensionless viscosity ratio $\beta$ and the couple stress constant $\eta^{\prime}$ on the micro-rotation velocity $N_{\theta }$ are represented in Figs. 4, 5, and 6. It is noted from these figures that the micro-rotation velocity $N_{\theta }$ increases by the increasing of $\overline{\gamma }$ while it decreases as $\beta$ increases. Fig. 6 shows that the micro-rotation velocity $N_{\theta }$ decreases by increasing $\eta^{\prime}$ in the interval r ∈ [0, 0.75], otherwise, namely, after r = 0.75, it has an opposite behavior, i.e., the behavior of $\eta^{\prime}$ in the interval r ∈ [0, 0.75], is an inversed manner of its behavior in the interval r ∈ [0.75,1.2].

**Figure 7:**
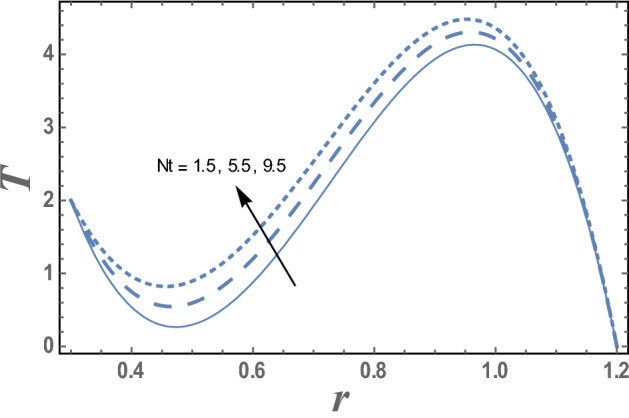
The variation of the temperature distribution T is plotted with r, for the different values of thermophoresis parameter Nt and for a system has particular values Pz = 1, γ = 0.7, α = 0.05, β = 10, M = 10, Da = 2, R = 1, Ec = 20, Pr = 1.5, Nb = 2.5, $\overline{\gamma }$ = 1.2, $\eta^{\prime}$ = 1, ${r}_{1}$ = 0.3, ${r}_{2}$ = 1.2, $\delta_{1}$ = 0.4.

**Figure 8:**
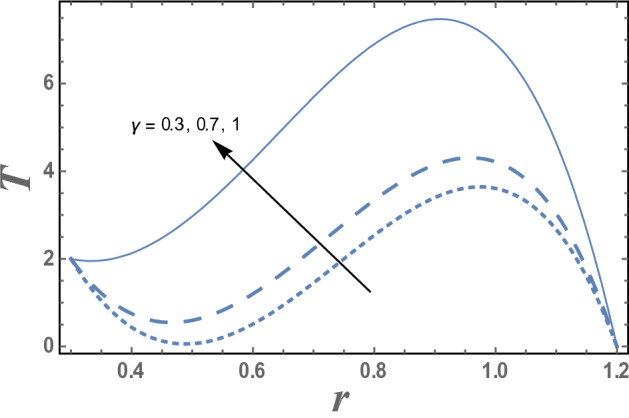
The variation of the temperature distribution T is plotted with r, for the different values of upper limit of apparent viscosity coefficient γ and for a system has particular values Pz = 1, α = 0.05, β = 10, M = 10, Da = 2, R = 1, Ec = 20, Pr = 1.5, Nt = 5.5, Nb = 2.5, $\overline{\gamma }$ = 1.2, $\eta^{\prime}$ = 1, ${r}_{1}$ = 0.3, ${r}_{2}$ = 1.2, $\delta_{1}$ = 0.4.

**Figure 9:**
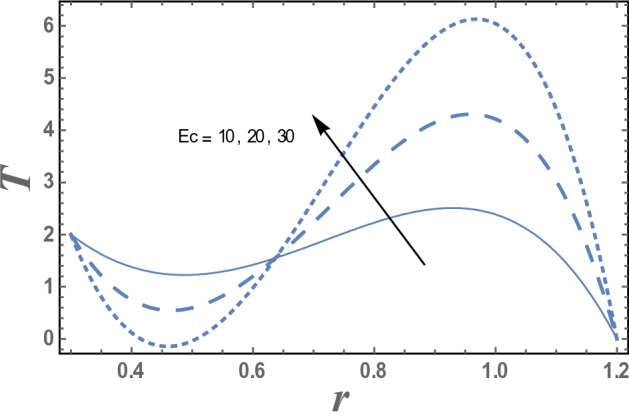
The temperature distribution T is plotted with r, for different values of Eckert number Ec and for a system has particular values Pz = 1, γ = 0.7, α = 0.05, $\beta$ = 10, M = 10, Da = 2, R = 1, Ec = 20, Pr = 1.5, Nt = 5.5, Nb = 2.5, $\overline{\gamma }$ = 1.2, $\eta^{\prime}$ = 1, ${r}_{1}$=0.3, ${r}_{2}$=1.2, $\delta_{1}$ = 0.4.

**Figure 10:**
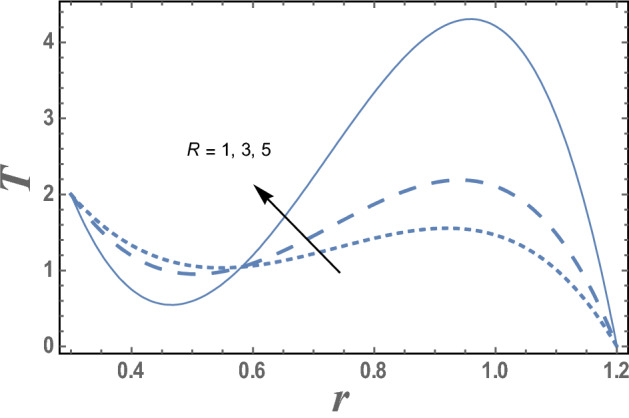
The variation of the temperature distribution T is plotted with r, for the different values of radiation parameter R and for a system has particular values Pz = 1, γ = 0.7, α = 0.05, β = 10, M = 10, Da = 2, Ec = 20, Pr = 1.5, Nt = 5.5, Nb = 2.5, $\overline{\gamma }$ = 1.2, $\eta^{\prime}$ = 1, ${r}_{1}$ = 0.3, ${r}_{2}$ = 1.2, $\delta_{1}$ = 0.4.

Figures 7 and 8 give the effects of thermophoresis parameter $Nt$ and the upper limit of apparent viscosity coefficient $\gamma$ on the temperature distribution T. It is seen from this figures that the temperature increases as $Nt$ increases while it decreases as $\gamma$ increases. The effects of Eckert number $Ec$ and radiation parameter R on the temperature distribution T are shown in Figs. 9 and 10, respectively. It observed from these figures that the effect of $Ec$ and R is similar to the effect of $\gamma$ and $Nt$ on T in the first interval, respectively. It is clear that the temperature is decreases by increasing $Ec$ and increases by increasing R till a value of r, after which it increases by increasing $Ec$ and decreases by increasing R. The effects of both M and Nb on the temperature distribution are found to be similar to effect of $Nt$ given in Fig. 7 while the effects of β is the same as $\gamma$ given in the Fig. 8.

**Figure 11:**
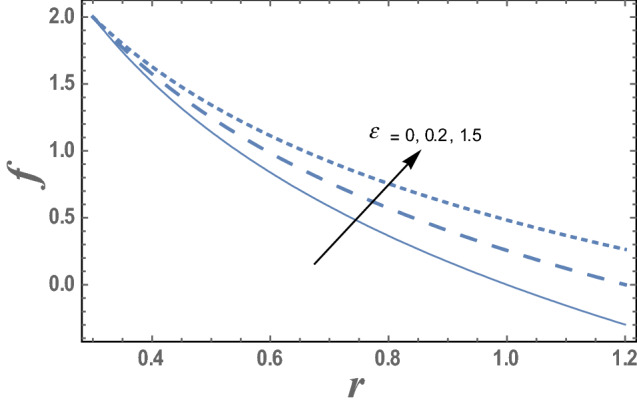
The variation of the nanoparticle distribution f is plotted with r, for the different values of the amplitude ratio ε and for a system has particular values Pz = 1, γ = 0.7, α = 0.05, β = 10, M = 10, Da = 2, R = 1, Ec = 20, Pr = 1.5, Nt = 5.5, Nb = 2.5, $\overline{\gamma }$ = 1.2, $\eta^{\prime}$ = 1, ${r}_{1}$ = 0.3, $\delta_{1}$ = 0.4.

**Figure 12:**
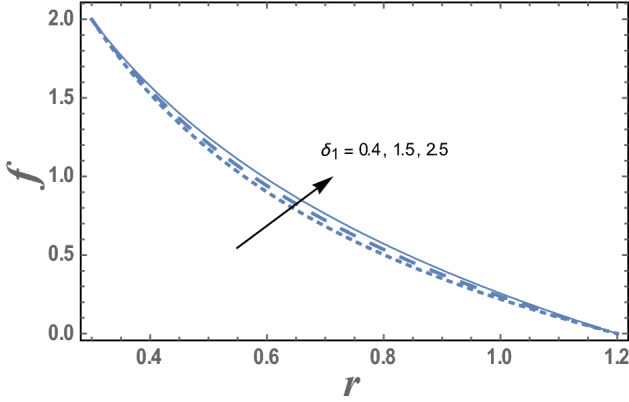
The variation of the nanoparticle distribution f is plotted versus r, different values of the chemical reaction parameter $\delta_{1}$ and for a system has particular values Pz = 1, γ = 0.7, α = 0.05, $\beta$ = 10, M = 10, Da = 2, R = 1, Ec = 20, Pr = 1.5, Nt = 5.5, Nb = 2.5, $\overline{\gamma }$ = 1.2, $\eta^{\prime}$ = 1, ${r}_{1}$=0.3, ${r}_{2}$=1.2, $\delta_{1}$ = 0.4.

The influence of the amplitude ratio $\varepsilon$ and the chemical reaction parameter $\delta_{1}$ on the nanoparticles concentration distribution are given in Figs. 11 and 12. It is obvious that the nanoparticles concentration increases as $\varepsilon$ increases while decreases as $\delta_{1}$ increases. This indicates that diffusion rates of nanoparticles are varied due to the effect endothermic chemical reaction. Chemical reaction is said to be endothermic if heat is absorbed. Hence, an increases of chemical reaction variable results in decreases of concentration.

**Figure 13:**
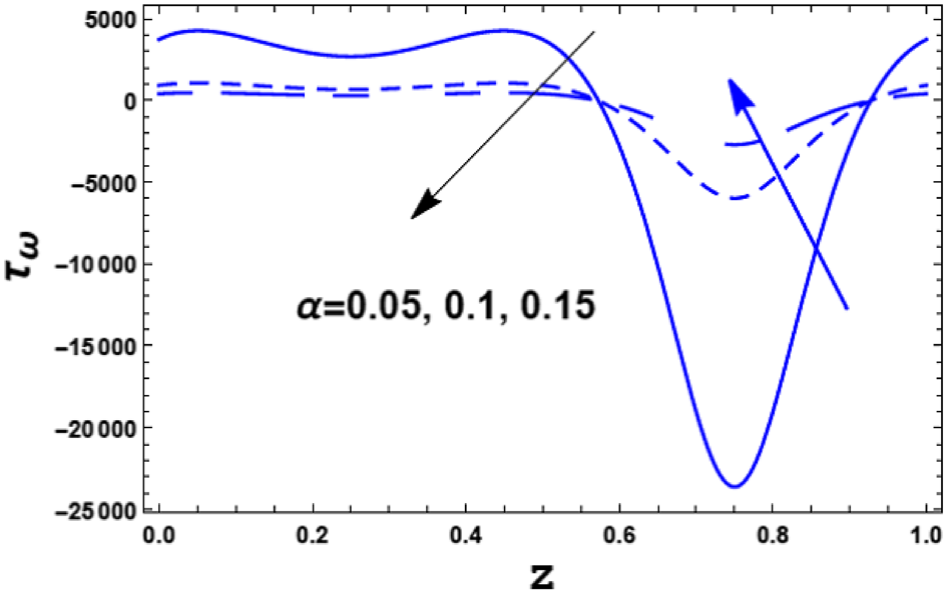
The variation of the skin friction $\tau_{\omega }$ is plotted with $z$, for the different values of the couple stress fluid parameter $\alpha$.

**Figure 14:**
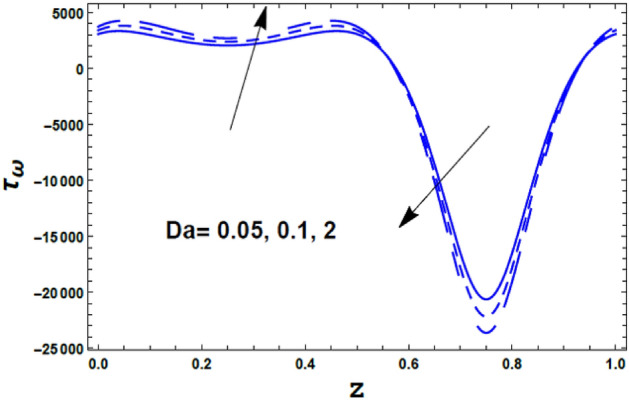
The variation of the skin friction $\tau_{\omega }$ is plotted with $z$, for the different values of the Darcy number $Da$.

Figure 13 illustrates the effect of the couple stress parameter $$\alpha$$ on the skin friction coefficient $$\tau_{\omega } \left( z \right).$$ It is found that the skin friction coefficient $$\tau_{\omega }$$ has a dual behavior under the influence of the couple stress parameter $$\alpha .$$ Hence, it decreases with an enrichment in the value of the couple stress parameter $$\alpha$$ along the interval $$\alpha \in$$$$\left[ {0,0.6} \right]$$. Meanwhile, along the interval $$\alpha \in$$$$\left[ {0.61,0.9} \right]$$ the inverse behavior occurred. Figure 14 shows the effect of the Darcy number $$Da$$ on the skin friction coefficient $$\tau_{\omega } \left( z \right).$$ It is noticed that the Darcy number $$Da$$ has an opposite effect when compared with the couple stress parameter $$\alpha .$$

**Figure 15:**
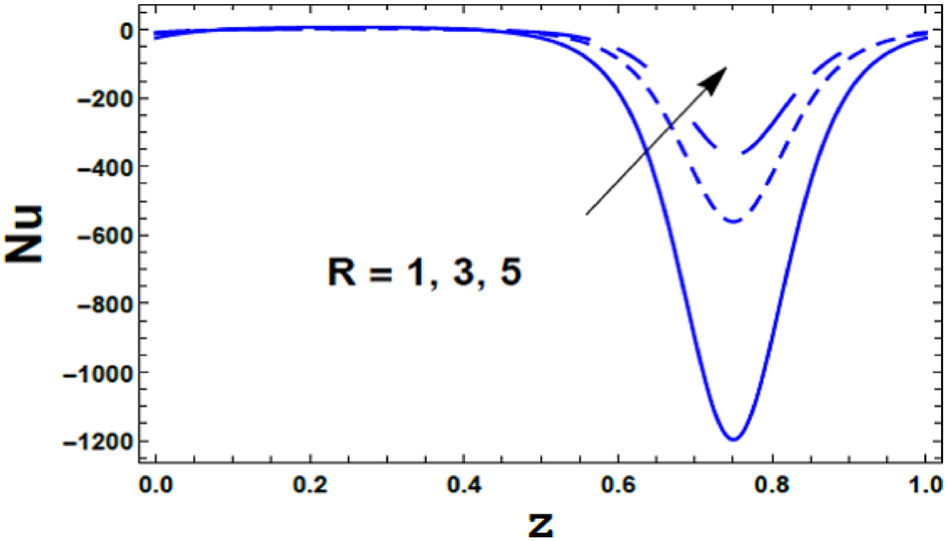
The variation of Nusselt number $Nu$ is plotted versus $z$, different values of the radiation parameter $R$.

**Figure 16:**
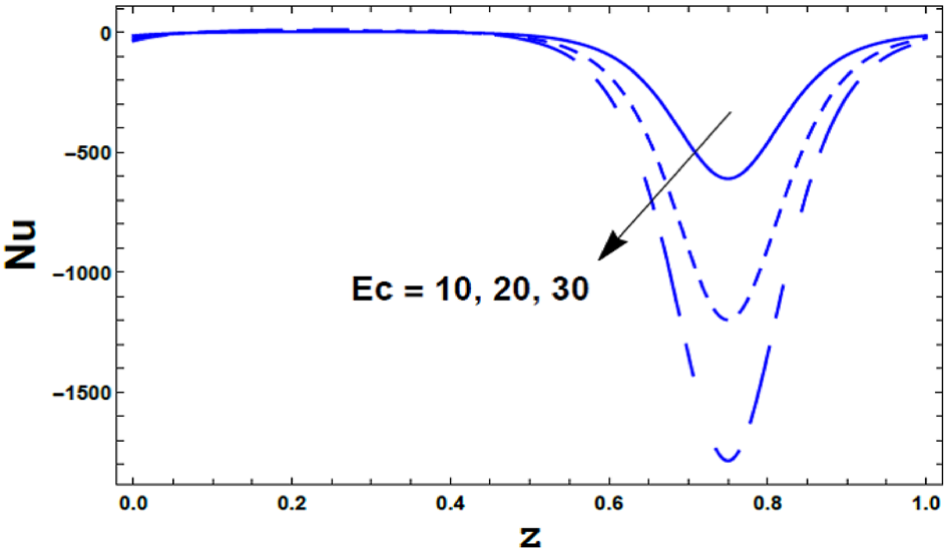
The variation of Nusselt number $Nu$ is plotted versus $z$, different values of the Eckert number $Ec$.

Moreover, the effect of the radiation parameter $R$ on the Nusselt number $Nu\left( z \right)$ is depicts in Fig. 15. As shown from this figure, the Nusselt number $Nu\left( z \right)$ increases with an increasing in the value of the radiation parameter $R$. Meanwhile, as seen from Fig. 16 the Nusselt number $Nu\left( z \right)$ decreases with an enlargement in the value of the Eckert number $Ec$.

**Figure 17:**
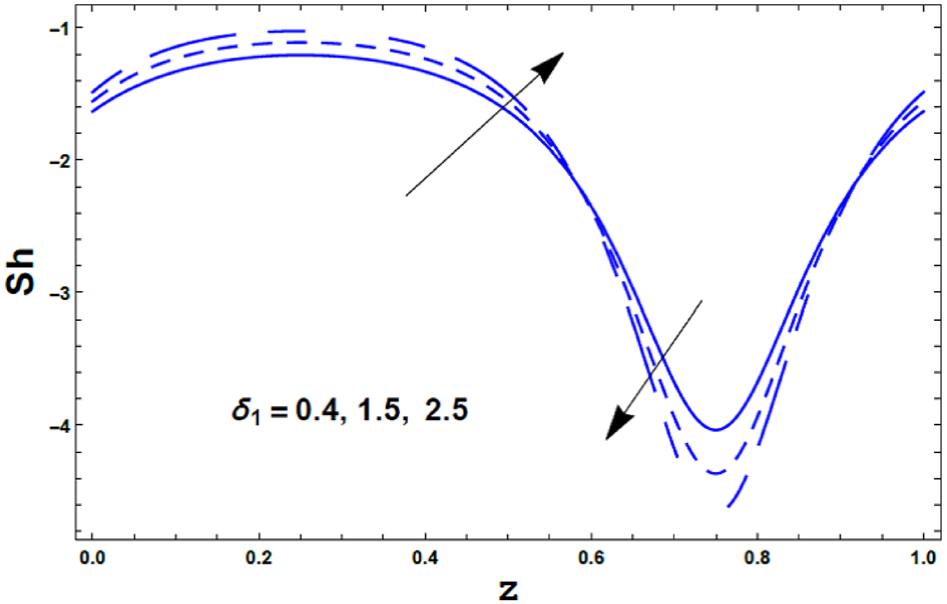
The variation of Sherwood number $Sh$ is plotted versus $z$, different values of the chemical reaction parameter $\delta_{1}$.

**Figure 18:**
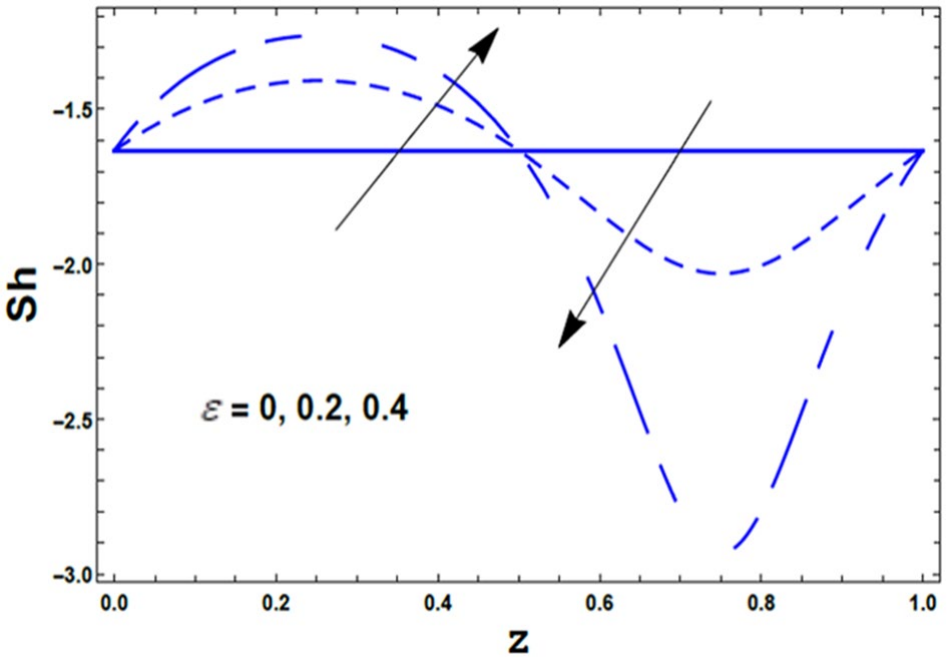
The variation of Sherwood number $Sh$ is plotted versus $z$, different values of the amplitude ratio *ε*.

Finally, the effects of the chemical reaction parameter $$\delta_{1}$$ and the amplitude ratio parameter $$\varepsilon$$ on the nano Sherwood number $$Sh\left( z \right)$$ are displayed through Figs. 17 and 18. As noticed from these figures, the nano Sherwood number $$Sh\left( z \right)$$ has a dual behavior under the influences of both $$\delta_{1}$$ and $$\varepsilon$$. Thus, it enhances with an enrichment in the value of the chemical reaction parameter $$\delta_{1}$$ along the interval $$\delta_{1} \in$$$$\left[ {0,0.6} \right]$$. However, along the interval $$\delta_{1} \in$$$$\left[ {0.61,0.9} \right]$$ the vis versa occurred. Also, the nano Sherwood number $$Sh\left( z \right)$$ increases with an increasing in the amplitude ratio parameter $$\varepsilon$$ along the interval $$\varepsilon \in$$$$\left[ {0,0.55} \right]$$. However, along the interval $$\varepsilon \in$$$$\left[ {0.6,1.0} \right]$$ the vis versa happened.

The effect of the micro-rotation parameter $\overline{\gamma }$ on the micro-rotation velocity N as function of the radial coordinate r is shown in Fig. 4 and for a system has particular values Pz = 1, γ = 0.7, α = 0.05, $\beta$ = 10, M = 10, Da = 2, R = 1, Ec = 20, Pr = 1.5, Nt = 5.5, Nb = 2.5, $\overline{\gamma }$ = 1.2, $\eta^{\prime}$ = 1, ${r}_{1}$=0.3, ${r}_{2}$=1.2, $\delta_{1}$ = 0.4.

The effect of the dimensionless viscosity ratio $\beta$ on the micro-rotation velocity N as a function of the radial coordinate r is shown in Fig. 5 and for a system has particular values Pz = 1, γ = 0.7, α = 0.05, $\beta$ = 10, M = 10, Da = 2, R = 1, Ec = 20, Pr = 1.5, Nt = 5.5, Nb = 2.5, $\overline{\gamma }$ = 1.2, $\eta^{\prime}$ = 1, ${r}_{1}$=0.3, ${r}_{2}$=1.2, $\delta_{1}$ = 0.4.

The effects of thermophoresis parameter Nt on the temperature distribution T is shown in Fig. 7 and for a system has particular values Pz = 1, γ = 0.7, α = 0.05, $\beta$ = 10, M = 10, Da = 2, R = 1, Ec = 20, Pr = 1.5, Nt = 5.5, Nb = 2.5, $\overline{\gamma }$ = 1.2, $\eta^{\prime}$ = 1, ${r}_{1}$=0.3, ${r}_{2}$=1.2, $\delta_{1}$ = 0.4.

The variation the temperature distribution with the radial coordinate r for different values of upper limit of apparent viscosity coefficient $\gamma$ is shown in Fig. 8 and for a system has particular values Pz = 1, γ = 0.7, α = 0.05, $\beta$ = 10, M = 10, Da = 2, R = 1, Ec = 20, Pr = 1.5, Nt = 5.5, Nb = 2.5, $\overline{\gamma }$ = 1.2, $\eta^{\prime}$ = 1, ${r}_{1}$=0.3, ${r}_{2}$=1.2, $\delta_{1}$ = 0.4.

The effect of radiation parameter R on the temperature T as a function of r of radial coordinate is shown in Fig. 10 and for a system has particular values Pz = 1, γ = 0.7, α = 0.05, $\beta$ = 10, M = 10, Da = 2, R = 1, Ec = 20, Pr = 1.5, Nt = 5.5, Nb = 2.5, $\overline{\gamma }$ = 1.2, $\eta^{\prime}$ = 1, ${r}_{1}$=0.3, ${r}_{2}$=1.2, $\delta_{1}$ = 0.4.

The variation the nanoparticle distribution f with the radial coordinate r, for different values of the amplitude ratio $\varepsilon$ is shown in Fig. 11 and for a system has particular values Pz = 1, γ = 0.7, α = 0.05, $\beta$ = 10, M = 10, Da = 2, R = 1, Ec = 20, Pr = 1.5, Nt = 5.5, Nb = 2.5, $\overline{\gamma }$ = 1.2, $\eta^{\prime}$ = 1, ${r}_{1}$=0.3, ${r}_{2}$=1.2, $\delta_{1}$ = 0.4.

## Trapping phenomenon

As usual in the hydrodynamic theory, for incompressible fluids in two–dimension, we may consider a stream function $\psi {\text{(r,z)}}$, which is defined as:

34 $${\text{u = }}\frac{{1}}{{\text{r}}}\left( {\frac{\partial \psi }{{\partial {\text{z}}}}} \right)\;{\text{and}}\;{\text{w = }} - \frac{{1}}{{\text{r}}}\left( {\frac{\partial \psi }{{\partial r}}} \right),$$

35 $$\begin{aligned} \psi (r,z) = & \frac{{a_{8} r^{2} }}{4} + \frac{{a_{7} r^{4} }}{{16}} + \frac{{a_{2} r^{5} }}{{25}} - \frac{{a_{3} r^{5} }}{5} - \frac{{a_{1} r^{6} }}{6} - \frac{{a_{4} r^{{5 + \eta ^{\prime}}} }}{{5 + \eta ^{\prime}}} - \frac{{a_{5} r^{{7 + \eta ^{\prime}}} }}{{7 + \eta ^{\prime}}} \\ & \quad - \frac{{r^{{3 + \eta ^{\prime}}} }}{{(3 + \eta ^{\prime})\left( {r_{1}^{{1 + \eta ^{\prime}}} - r_{2}^{{1 + \eta ^{\prime}}} } \right)}} - \frac{1}{5}a_{2} r^{5} Log[r] - \frac{1}{4}r^{4} \left( {a_{6} + a_{7} {\kern 1pt} Log[r]} \right) \\ & \quad + \frac{{r^{2} \left( {(1 - a_{9} )r_{1}^{{1 + \eta ^{\prime}}} + a_{9} r_{2}^{{1 + \eta ^{\prime}}} - a_{8} \left( {r_{1}^{{1 + \eta ^{\prime}}} - r_{2}^{{1 + \eta ^{\prime}}} } \right)Log[r]} \right)}}{{2\left( {r_{1}^{{1 + \eta ^{\prime}}} - r_{2}^{{1 + \eta ^{\prime}}} } \right)}} \\ \end{aligned}$$

Trapping is considered as an interesting phenomenon correlated with peristaltic transport. Trapping happens only in particular circumstances. Which depicted by a large amplitude ratio. In the wave frame of reference, asset of closed streamlines can be recognized in the most stretched region of the tube. The set of streamlines designated as a bolus of fluid. This bolus transports with the wave in the laboratory frame. There is an inner circulation which can recognize inside the bolus.

**Figure 19:**
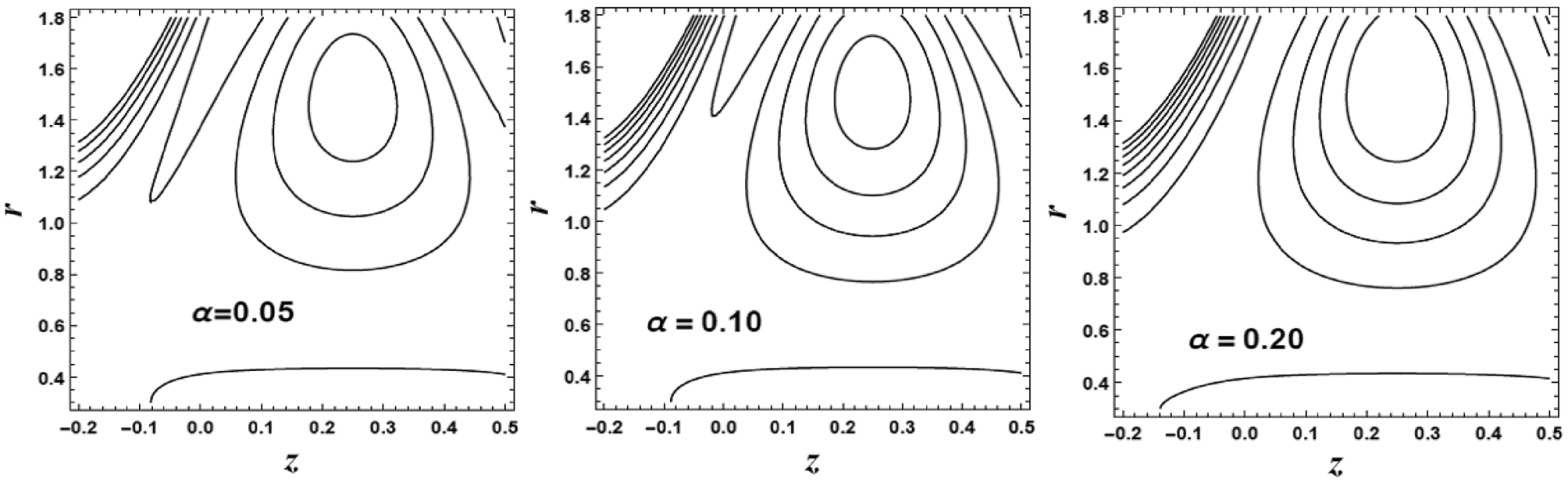
The streamlines contour is plotted for different values of *α*.

**Figure 20:**
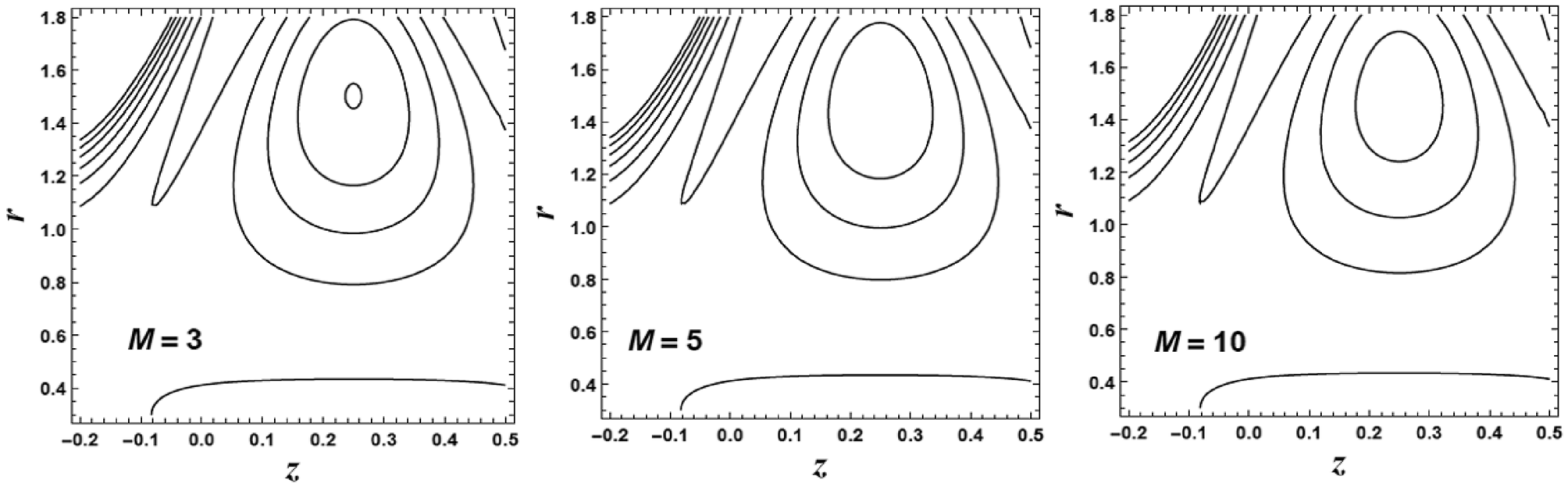
The streamlines contour is plotted for different values of *M*.

The circulation and size of the trapped bolus are displayed through Fig. 19. This figure is portrayed to reflects the features of the couple stress parameter $\alpha$ on the streamlines. It is depicted that the bolus increases in size with an enlarge in the value of the couple stress parameter $\alpha$. Also, the number of the circulations is enlarged. Figure 20 displays the circulation and size of the trapped bolus for different values of the magnetic parameter $M$ on the streamlines. It is noticed that the bolus decreases in size with an enhancement in the value of the magnetic parameter *M*. Also, the number of the circulations is decreased.

## Conclusion

In this article, the MHD peristaltic flow of a couple stress with heat transfer of micropolar biviscosity nanofluid is studied. We assumed that long wavelength and low-Reynolds number approximations to simplify the system of the nonlinear partial differential equations. The effects of porous medium, chemical reaction and radiation are taken into consideration. This problem is an extension the problem of Eldabe and Abouzeid^22^ and Abouzeid^5^. Some figures are drawn to show the effect of the different non-dimensional parameters on the axial velocity w, microrotation velocity N, temperature T and nanoparticles concentration distributions f. Furthermore, the values of the skin friction coefficient, Nusselt and nano Sherwood numbers are computed and presented graphically through some draws. Moreover, the trapping phenomenon is discussed throughout a set of figures.

1. The axial velocity w increases with the increase each of α,$\gamma$ and M, whereas it decreases as Da, β, $\eta^{\prime}$ and ε increase.
2. As Nb, Nt and M increase, the temperature T increases, while it decreases with the increase of β and $\gamma$
3. The micro-rotation velocity increase as $\overline{\gamma }$ increases, while it decreases as β and ε increases.
4. The nanoparticles phenomena increases as ε increases, whereas it decreases as $\delta_{1}$ increases.
5. The size of the trapped bolus is increased with the elevation in the value of the couple stress parameter α.
6. The size of the trapped bolus is reduced with the increasing in the value of the magnetic parameter *M*.

## Data Availability

The datasets generated and/or analyzed during the current study are not publicly available due [All the required data are only with the corresponding author] but are available from the corresponding author on reasonable request.

## References

[1] Abouzeid MY (2019). Implicit homotopy perturbation method for mhd non-Newtonian nanofluid flow with Cattaneo-Christov heat flux due to parallel rotating disks. J. Nanofluids.

[2] El-dabe NT, Abouzeid MY, Ahmed OS (2020). Motion of a thin film of a fourth grade nanofluid with heat transfer down a vertical cylinder: Homotopy perturbation method application. J. Adv. Res. Fluid Mech. Thermal Sci..

[3] El-dabe NT, Abouzeid MY (2018). Radially varying magnetic field effect on peristaltic motion with heat and mass transfer of a non-Newtonian fluid between two co-axial tubes. Therm. Sci..

[4] Akram S, Razia A, Afzal F (2020). Effects of velocity second slip model and induced magnetic field on peristaltic transport of non-Newtonian fluid in the presence of double-diffusivity convection in nanofluids. Arch. Appl. Mech..

[5] Abouzeid MY (2018). Homotopy perturbation method for couple stresses effect on MHD peristaltic flow of a non-Newtonian nanofluid. Microsyst. Technol..

[6] Ismael A, Eldabe N, Abouzeid M, Elshabouri S (2022). Entropy generation and nanoparticles cu o effects on mhd peristaltic transport of micropolar non-newtonian fluid with velocity and temperature slip conditions. Egypt. J. Chem..

[7] Ouaf ME, Abouzeid MY, Younis YM (2022). Entropy generation and chemical reaction effects on MHD non-Newtonian nanofluid flow in a sinusoidal channel. Int. J. Appl. Electromagnet Mech.

[8] Akhtar S, McCash LB, Nadeem S, Saleem S, Issakhov A (2021). Convective heat transfer for Peristaltic flow of SWCNT inside a sinusoidal elliptic duct. Sci. Prog..

[9] Eldabe NT, Elshabouri S, Elarabawy H, Abou zeid MY, Abuiyada A (2022). Wall properties and Joule heating effects on MHD peristaltic transport of Bingham non-Newtonian nanofluid. Int. J. Appl. Electromag. Mech..

[10] Das S, Barman SB, Jana RN, Makinde OD (2021). Hall and ion slip currents’ impact on electromagnetic blood flow conveying hybrid nanoparticles through an endoscope with peristaltic waves. BioNanoScience.

[11] El-dabe, N. T., Abou-zeid, M. Y., Abosaliem, A., Alana, A., Hegazy, N. Homotopy perturbation approach for Ohmic dissipation and mixed convection effects on non-Newtonian nanofluid flow between two co-axial tubes with peristalsis. *J. Appl. Electromagn. Mech.* **67**, 153–163 (2021)

[12] Ibrahim M, Abdallah N, Abouzeid M (2022). Activation energy and chemical reaction effects on MHD Bingham nanofluid flow through a non-Darcy porous media. Egypt. J. Chem..

[13] Akhtar S (2022). Analytical solutions of PDEs by unique polynomials for peristaltic flow of heated Rabinowitsch fluid through an elliptic duct. Sci. Rep..

[14] Gudekote M (2020). Influence of variable viscosity and wall properties on the peristalsis of Jeffrey fluid in a curved channel with radial magnetic field. Int. J. Therm. Sci. Technol..

[15] Mansour HM, Abouzeid MY (2019). Heat and mass transfer effect on non-newtonian fluid flow in a non-uniform vertical tube with peristalsis. J. Adv. Res. Fluid Mech. Therm. Sci..

[16] Eldabe NTM, Abouzeid MY, Ali HA (2020). Effect of heat and mass transfer on Casson fluid flow between two co-axial tubes with peristalsis. J. Adv. Res. Fluid Mech. Therm. Sci..

[17] Abouzeid MY, Mohamed MAA (2017). Homotopy perturbation method for creeping flow of non-Newtonian Power-Law nanofluid in a nonuniform inclined channel with peristalsis. Z. Naturforsch.

[18] Eldabe NTM, Rizkallah RR, Abouzeid MY, Ayad VM (2022). Effect of induced magnetic field on non-Newtonian nanofluid Al_2_IO_3_ motion through boundary-layer with gyrotactic microorganisms. Therm. Sci..

[19] Eldabe NTM, Abouzeid MY, Elshabouri SM, Salama TN, Ismael AM (2022). Ohmic and viscous dissipation effects on micropolar non-Newtonian nanofluid Al_2_O_3_ flow through a non-Darcy porous media. Int. J. Appl. Electromag. Mech..

[20] Akhtar S, Almutairi S, Nadeem S (2022). Impact of heat and mass transfer on the Peristaltic flow of non-Newtonian Casson fluid inside an elliptic conduit: Exact solutions through novel technique. Chin. J. Phys..

[21] Shamshuddin MD, Mishra SR, Bég OA, Kadir A (2019). Unsteady reactive magnetic radiative micropolar flow, heat and mass transfer from an inclined plate with Joule heating: a model for magnetic polymer processing. Proc. IMechE- Part C. Mech. Eng. Sci..

[22] Eldabe NTM, Abouzeid MY (2014). Magnetohydrodynamic peristaltic flow with heat and mass transfer of micropolar biviscosity fluid through a porous medium between two co-axial tubes. Arab. J. Sci. Eng..

[23] Eldabe NT, Abouzeid MY (2017). Homotopy perturbation method for MHD pulsatile non-Newtonian nanofluid flow with heat transfer through a non-Darcy porous medium. J. Egypt. Math. Soc..

[24] Beg OA (2021). Unsteady nonlinear magnetohydrodynamic micropolar transport phenomena with Hall and Ion-slip current effects: Numerical study. Int. J. Appl. Electromagn. Mech..

[25] Kamran M, Wiwatanapataphee B (2018). Chemical reaction and Newtonian heating effects on steady convection flow of a micropolar fluid with second order slip at the boundary. Eur. J. Mech. B Fluids.

[26] Mohamed MA, Abou-zeid MY (2019). MHD peristaltic flow of micropolar Casson nanofluid through a porous medium between two co-axial tubes. J. Porous Media.

[27] El Ouaf M, Abouzeid M (2021). Electromagnetic and non-Darcian effects on a micropolar non-Newtonian fluid boundary-layer flow with heat and mass transfer. Int. J. Appl. Electromag. Mech..

[28] Eldabe NTM, Rizkallah RR, Abouzeid MY, Ayad VM (2020). Thermal diffusion and diffusion thermo effects of Eyring- Powell nanofluid flow with gyrotactic microorganisms through the boundary layer. Heat Transf. Asian Res..

[29] Reddy KV, Reddy MG, Makinde OD (2021). Heat and mass transfer of a peristaltic electro-osmotic flow of a couple stress fluid through an inclined asymmetric channel with effects of thermal radiation and chemical reaction. Periodica Polytechnica Mech. Eng..

[30] Abouzeid MY (2016). Dissipation on peristaltic flow of micropolar non-Newtonian nanofluid: Application of homotopy perturbation method. Results Phys..

[31] Bhatti MM, Zeeshan A, Asif MA, Ellahi R, Sadiq MS (2022). Non-uniform pumping flow model for the couple stress particle-fluid under magnetic effects. Chem. Eng. Commun..

[32] Eldabe NTM, Hassan MA, Abouzeid MY (2015). Wall properties effect on the peristaltic motion of a coupled stress fluid with heat and mass transfer through a porous media. J. Eng. Mech..

[33] Ellahi R, Bhatti MM, Fetecau C, Vafai K (2016). Peristaltic flow of couple stress fluid in a non-uniform rectangular duct having compliant walls. Commun. Theor. Phys..

[34] Zigta B (2020). Mixed convection on MHD flow with thermal radiation, chemical reaction and viscous dissipation viscous dissipation embedded in a porous medium. Int. J. Appl. Mech. Eng..

[35] Ramesh K, Devakar M (2015). Magnetohydrodynamic peristaltic transport of couple stress fluid through porous medium in an inclined asymmetric channel with heat transfer. J. Magn. Magn. Mater.

[36] Eldabe NTM, Hassan AA, Mohamed MAA (2003). Effect of couple stresses on the MHD of a non-newtonian unsteady flow between two parallel porous plates. Z. Naturforsch.

[37] Chakravarty S, Das S, Hadjesfandiari AR, Dargush GF (2018). variational inequalities for heterogeneous microstructures based on couple-stress theory. Int. J. Multiscale Comput. Eng..

[38] Jangili S, Adesanya SO, Ogunseye HA, Lebelo R (2019). Couple stress fluid flow with variable properties: A second law analysis. Math. Meth. Appl. Sci..

[39] Zeeshan A, Ali Z, Gorji MR, Hussain F, Nadeem S (2020). Flow analysis of biconvective heat and mass transfer of two-dimensional couple stress fluid over a paraboloid of revolution. Int. J. Mod. Phys. B.

[40] Vaidya H (2021). Combined effects of homogeneous and heterogeneous reactions on peristalsis of Ree-Eyring liquid: Application in hemodynamic flow. Heat Transf.-Asian Res..

[41] Vaidya H (2020). Heat and mass transfer analysis of MHD peristaltic flow through a complaint porous channel with variable thermal conductivity. Physica Scripta.

[42] Vaidya H (2020). Peristaltic flow of non-Newtonian fluid through an inclined complaint nonlinear tube: Application to chyme transport in the gastrointestinal tract. Eur. Phys. J. Plus.

[43] Rohsenow WM, Hartnett JP, Cho YI (1998). Handbook of Heat Transfer.

